# Seroprevalence of *Toxoplasma gondii* and *Encephalitozoon cuniculi* among domestic rabbits in central China

**DOI:** 10.1051/parasite/2018010

**Published:** 2018-03-09

**Authors:** Shuai Wang, Zhijun Yao, Lingjuan Li, Yaoqian Pan, Pengju Li, Xiaoxu Nan, Qing Xie, Zhenchao Zhang

**Affiliations:** 1 School of Basic Medical Sciences, Xinxiang Medical University, Xinxiang, Henan, 453003 PR China; 2 Henan Muxiang Veterinary Pharmaceutical Co., Ltd, Zhengzhou, Henan, 450000 PR China; 3 School of Life Science and Technology, Xinxiang University, Xinxiang, Henan, 453003 PR China; 4 School of Stomatology, Xinxiang Medical University, Xinxiang, Henan, 453003 PR China

**Keywords:** *Toxoplasma gondii*, *Encephalitozoon cuniculi*, domestic rabbit, seroprevalence, central China

## Abstract

Rabbits (*Oryctolagus cuniculus*) are frequently reared for meat production in China. The aim of this study was to assess the seroprevalence of *Toxoplasma gondii* and *Encephalitozoon cuniculi*, and risk factors of infection in domestic rabbits raised in Henan province, central China. 1,213 serum samples of domestic rabbits were collected and tested for anti-*T. gondii* and anti-*E. cuniculi* antibodies using a modified agglutination test (MAT) and an enzyme-linked immunosorbent assay (ELISA), respectively. The serum positive rates of *T. gondii* and *E. cuniculi* were 128/1,213 (10.55%) and 235/1,213 (19.37%), respectively. Co-infection of *T. gondii* and *E. cuniculi* was demonstrated in 84 specimens; 44 rabbits were seropositive for *T. gondii* alone, while 151 rabbits were seropositive for *E. cuniculi* alone. The main risk factors simultaneously associated with *T. gondii* and *E. cuniculi* infection were the age of the rabbit, the type of food, and the rabbit rearing system. Serum positive rates of *T. gondii* and *E. cuniculi* among domestic rabbits were high, indicating the possibility of public health issues.

## Abstract (fr)

Les lapins (*Oryctolagus cuniculus*) sont fréquemment élevés pour la production de viande en Chine. Le but de l’étude était de déterminer la séroprévalence de *Toxoplasma gondii* et d’*Encephalitozoon cuniculi* ainsi que les facteurs de risque d’infection chez les lapins domestiques élevés dans la province du Henan, en Chine centrale. 1 213 échantillons de sérum de lapins domestiques ont été collectés et testés pour les anticorps anti-*T. gondii* et anti-*E. cuniculi* en utilisant un test d’agglutination modifié (MAT) et un dosage immuno-enzymatique (ELISA), respectivement. Les taux sériques positifs de *T. gondii* et *E. cuniculi* étaient de 128/1 213 (10,55%) et de 235/1 213 (19,37%), respectivement. La co-infection par *T. gondii* et *E. cuniculi* a été démontrée chez 84 spécimens ; 44 lapins étaient séropositifs pour *T. gondii* seul, alors que 151 lapins étaient séropositifs pour *E. cuniculi* seul. Les principaux facteurs de risque associés simultanément à l’infection par *T. gondii* et *E. cuniculi* étaient l’âge du lapin, le type d’aliment et le système d’élevage du lapin. Les taux sériques positifs de *T. gondii* et d’E*. cuniculi* chez les lapins domestiques étaient élevés, ce qui indique la possibilité de problèmes de santé publique.

## Introduction

*Toxoplasma gondii* (*T. gondii*) is one of the most common universal zoonotic protozoan parasites, ubiquitous throughout the world, and widely prevalent in humans and animals [8]. It is estimated that up to one-third of the world’s human population has been infected with *T. gondii* [10]. Almost all warm blooded animals are infected by *T. gondii*, including rabbits [7]. The main route for humans to acquire *T. gondii* infection is the consumption of raw or undercooked meat containing tissue cysts from intermediate hosts [8].

The transmission mode of *T. gondii* to rabbits is through water or food containing oocysts from feline excrement, or through placenta from pregnant females to offspring [8]. Humans may become infected not only by eating undercooked rabbit meat but also from contaminated hand–to-mouth processes after slaughtering, skinning rabbits or dealing with undercooked or raw rabbit meat [4,9].

*Encephalitozoon cuniculi* (*E. cuniculi*) is a common pathogen of rabbits causing chronic renal and central nervous system disease characterized by granuloma formation and fibrosis [16]. However, it also affects other mammals, including rodents, herbivores, carnivores, nonhuman primates, and humans [1,6]. Infected rabbits eliminate the spores in urine and feces. Humans become infected principally by intake of water or food contaminated with infective spores. Human encephalitozoonosis is mostly found in immunocompromised patients, including organ transplant recipients, HIV patients, and cancer patients who are being treated with chemotherapy [2]. In many rabbits, the infection can persist subclinically for a long period of time [16].

In China, rabbits are frequently reared for meat production and are consequently considered a possible source of infection by *T. gondii* and *E. cuniculi* in humans. However, little is known about natural infection of *T. gondii* and *E. cuniculi* in domestic rabbits in China. Therefore, this study was conducted to investigate the seroprevalence and the risk factors related to seropositivity of *T. gondii* and *E. cuniculi* among domestic rabbits in Henan province, central China. The results will lay the groundwork for controlling both *T. gondii* and *E. cuniculi* infections among domestic rabbits in central China.

## Material and methods

### Ethical statements

All protocols in this study were reviewed and approved by the Ethics Committee of the Xinxiang Medical University (reference no. 2015018).

### The study site

**Figure 1:**
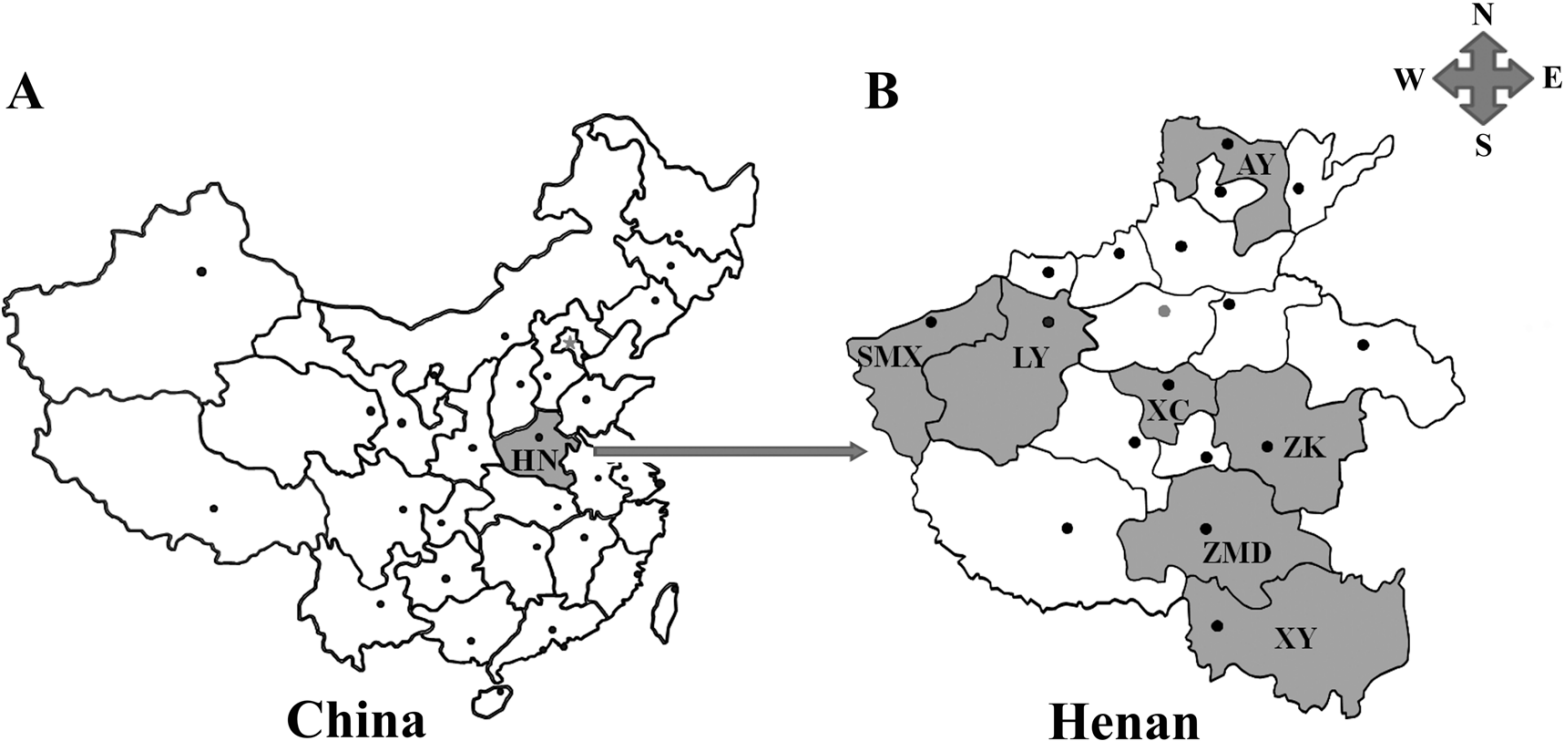
Geographic distribution of the sampling sites in Henan province, central China used in this study. A: Henan province (HN, shadowed areas) is located in the central part of mainland China. B: Shadowed areas are the sampling locations for the present survey. AY: Anyang; SMX: Sanmenxia; LY: Luoyang; XC: Xuchang; ZK: Zhoukou; ZMD: Zhumadian; XY: Xinyang.

The study was conducted in Henan province, which is situated in the middle section of China with an approximate population of 106.01 million and total surface area of 167,000 km^2^. The Yellow River flows through the middle section of Henan, which is seated within north latitude of 31°23′−36°22′ and east longitude of 110°21′−116°39′. Due to the mainland monsoon type climate, four seasons are distinct with the year-round average temperature of 12.1-15.7 °C and year-round average precipitation of 532.5-1380.6 mm. Henan province contains seventeen cities and Zhengzhou is the capital city. Seven cities which include Anyang (35°13′−36°22′N, 113°37′−114°58′E), Sanmenxia (33°31′−35°05′N, 110°21′−112°01′E), Luoyang (33°35′−35°05′N, 111°08′−112°59′E), Xuchang (33°16′−34°24′N, 113°03′−114°19′E), Zhumadian (32°18′−33°35′N, 113°10′−115°12′E), Xinyang (31°46′−31°52′N, 114°01′−114°06′E) and Zhoukou (33°03′−34°20′N, 114°05′−115°39′E), located in the northern, western, central, southern and eastern parts of Henan province (Fig. 1), were selected for sample collections. All of the above places account for most rabbit meat supplies to districts in and around Henan.

### Collecting samples

A total of 1213 blood specimens of domestic rabbits were collected from the seven above-mentioned cities within Henan province during the period from June 2015 to December 2016. Information on location, species, gender, ages of rabbits as well as the feeding conditions of respective rabbits was recorded. Serum specimens prepared by centrifuging whole blood were subsequently transferred into 1.5 ml Eppendorf tubes and preserved under the temperature of −80 °C before being tested against anti-*E. cuniculi* and *T*. *gondii* antibodies.

### Determining anti-*T*. *gondii* antibody

Based on previous studies, the anti-*T. gondii* antibodies in serum specimens were determined using the modified agglutination test (MAT) [8,13,21]. *T. gondii* whole cell antigen (formalin-fixed whole tachyzoites) prepared using the RH strain of *T. gondii* cultivated *via* human foreskin fibroblast cells was purchased from KeraFAST, Inc. (Boston, MA, USA). Briefly, serum samples were diluted by serum dilution buffer using 2-fold serial dilutions from 1:25 to 1:3,200. Fifty microlitres of diluted serum samples were used for agglutinating with 50 µl of antigen mixture (mixture of antigen dilution buffer, 2-mercaptoethanol, Evans blue dye solution, and *T. gondii* whole cell antigen) in a U-bottom 96-well microtiter plate under the temperature of 37 °C for a whole night. The formation of parasite agglutinating layers in wells which contained diluted serum specimens with a ratio of 1:25 or higher indicated positive results. Each assay contained negative and positive control groups.

### Determination of antibodies against *E. cuniculi*

The anti-*E. cuniculi* antibody levels of serum samples from domestic rabbits were verified and detected using a commercial *Encephalitozoon cuniculi* (EC) ELISA kit (Medicago, Uppsala, Sweden), following the manufacturer’s instructions.

### Statistical analysis

Chi square tests were used to analyse the variation of serum positive rates for *T*. *gondii* and *E. cuniculi* resulting from variates including species, gender, ages, the type of food, and the rearing system. Windows version SPSS 20 (SPSS Inc, Chicago, IL, USA) was used to perform all statistical analyses. A *p-*value lower than 0.05 was regarded as the threshold for statistically significant differences.

## Results

### Seroprevalence of *T. gondii*

As shown in Table 1, the overall recorded serum positive rate for *T. gondii* among rabbits from Henan province, central China was 10.55% (128/1213), with titers of 1:25 in 69, 1:50 in 36, 1:100 in 14, 1:200 in 5, 1:400 in 3, and 1:800 in 1. The serum positive rates of 7 sampling sites were within the range of 7.64% in Sanmenxia city to 14.06% in Luoyang city (Table 1). New Zealand rabbits exhibited the maximum value of 12.34%, followed by Chinese rabbits (10.73%) and then Japanese White rabbits (10.58%), while the prevalence found in Rex rabbits was 9.17%.

**Table 1:** Seroprevalence of *T. gondii* infection in domestic rabbits in Henan province, central China.

Variable	No. examined	No. positive	Prevalence (%)	X^2^	*p*-value
Region					
Anyang	165	14	8.48	5.383	0.496
Sanmenxia	157	12	7.64		
Luoyang	192	27	14.06		
Xuchang	169	18	10.65		
Zhoukou	176	21	11.93		
Zhumadian	183	17	9.29		
Xinyang	171	19	11.11		
Species					
Japanese White rabbit	189	20	10.58	1.983	0.576
Rex rabbit	447	41	9.17		
New Zealand rabbit	316	39	12.34		
Chinese rabbit	261	28	10.73		
Gender					
Male	534	61	11.42	0.767	0.381
Female	679	67	9.87		
Age (months)					
≤6	316	21	6.65	14.361	0.001
6∼12	692	72	10.40		
≥12	205	35	17.07		
Food					
Feed	488	33	6.76	17.445	< 0.001
Fruits/vegetables/grains	319	51	15.99		
Mixed*	406	44	10.84		
Presence of cats					
Yes	743	93	12.52	7.840	0.005
No	470	35	7.45		
Rearing system					
Commercial farms	549	33	6.01	21.914	< 0.001
Household farms	664	95	14.31		
Total	1213	128	10.55		

* Mixture of feed and fruits, vegetables, and grains.

The rate of antibodies to *T. gondii* in male rabbits was 11.42% (61/534) and in female rabbits 9.87% (67/679) (Table 1). Although the seroprevalence in males was higher than the females, the difference was not significant (*p*>0.05). The prevalence of *T. gondii* infection in rabbits increased significantly (*p* < 0.01) with age. Rabbits no younger than twelve months exhibited the highest seroprevalence (17.07%), followed by rabbits of 6-12 months (10.40%), and then rabbits ≤ 6 months (6.65%) (Table 1).

The highest seroprevalence of *T. gondii* infection (15.99%) was found in rabbits fed with a mixture of fruits, vegetables or grains. The probability of rabbits being infected by *T. gondii* was increased by the presence of cats at the feeding farm compared to farms without cats (12.52% *vs* 7.45%, *p* < 0.01). In terms of rearing systems, the *T. gondii* seroprevalence in rabbits raised on commercial farms (6.01%) was significantly lower than that of animals raised on household farms (14.31%, *p* < 0.01).

The univariate analyses on the correlation between serum positive rates of *T. gondii* and possible risk factors are shown in Table 1, indicating significant associations between serum positive rates of *T. gondii* and food type, rabbit ages, cat presence on feeding farms, as well as the rearing system.

### Seroprevalence of *E. cuniculi*

As shown in Table 2, in total 235 (19.37%) rabbits were positive for anti-*E. cuniculi* antibodies. Rabbits with positive antibodies in serum were found at all tested sites, varying from 11.83% in Xuchang city to 29.55% in Zhoukou city (Table 2). Rex rabbits exhibited the highest value of serum positive rates in all studied species (24.16%), followed by New Zealand rabbits (17.41%), then Japanese White rabbits (16.93%), while the prevalence found in Chinese rabbits was 15.32%. The serum positive rate of female rabbits (21.50%) was remarkably higher than that of male rabbits (16.67%) (*p* < 0.05).

**Table 2:** Seroprevalence of *E. cuniculi* infection in domestic rabbits in Henan province, central China.

Variable	No. examined	No. positive	Prevalence (%)	X^2^	*p*-value
Region					
Anyang	165	31	18.79	28.003	< 0.001
Sanmenxia	157	33	21.02		
Luoyang	192	48	25.00		
Xuchang	169	20	11.83		
Zhoukou	176	52	29.55		
Zhumadian	183	24	13.11		
Xinyang	171	27	15.79		
Species					
Japanese White rabbit	189	32	16.93	10.803	0.013
Rex rabbit	447	108	24.16		
New Zealand rabbit	316	55	17.41		
Chinese rabbit	261	40	15.32		
Gender					
Male	534	89	16.67	4.475	0.034
Female	679	146	21.50		
Age (months)					
≤6	316	39	12.34	24.159	< 0.001
6∼12	692	135	19.51		
≥12	205	61	29.76		
Food					
Feed	488	56	11.48	36.364	< 0.001
Fruits/vegetables/grains	319	89	27.89		
Mixed*	406	90	22.17		
Rearing system					
Commercial farms	549	71	12.93	26.636	< 0.001
Household farms	664	164	24.70		
Total	1213	235	19.37		

* Mixture of feed and fruits, vegetables, and grains.

Moreover, it was revealed by comparing infection rates by ages that 39/316 *E. cuniculi* seropositive animals (12.34%) were young (≤ 6 months), 135/692 (19.51%) were adults (6∼12 months), and 61/205 (29.76%) were in older age groups (≥ 12 months). The *E. cuniculi* seropositivity of rabbits increased significantly (*p* < 0.01) with the increase of rabbit’s age.

Like in *T. gondii* infection, the highest *E. cuniculi* seroprevalence (27.89%) was found in rabbits fed with a mixture of fruits, vegetables or grains. Moreover, *E. cuniculi* seroprevalence in rabbits raised on commercial farms was also significantly lower than that of animals raised on household farms (12.93% *vs* 24.70%, *p* < 0.01).

### Co-infection with *T. gondii* and *E. cuniculi*

Among the 279 infected rabbits, co-infection with *E. cuniculi* and *T. gondii* was demonstrated in 84 specimens (30.11%). Forty-four rabbits (15.77%) were seropositive for *T. gondii* alone, while 151 rabbits (54.12%) were seropositive for *E. cuniculi* alone (Table 3).

**Table 3:** Frequency of single infection and co-infection in all 279 infected rabbits.

Infection	No. positive	Percentage (%)
*T. gondii* only	44	15.77
*E. cuniculi* only	151	54.12
Co-infection of *T. gondii* and *E. cuniculi*	84	30.11
Total	279	100.00

## Discussion

The current investigation showed that the total serum positive rate of *T. gondii* was 10.55% among domestic rabbits in Henan, central China, which was lower than that found in Shanghai (23.4%) [22], but higher than that observed in Liaoning (6.5%), Jilin (4.5%), Heilongjiang (3.7%), and the Inner Mongolia Autonomous Region (3.5%) [13] of China. Meanwhile, the serological results of this study revealed that the overall serum positive rate for *E. cuniculi* was 19.37% in domestic rabbits in Henan. In comparison to other provinces within China mainland, the serum positive rate of 19.37% was lower than the values of 22.2% among rabbits investigated previously in Inner Mongolia, 30.9% in Liaoning [13], and 41.0% in Jilin [15], but higher than that observed in Sichuan (9.0%), Chongqing (6.0%) [15], and Heilongjiang (17.3%) [13]. The variations of serum positive rates for anti-*T. gondii* and anti-*E. cuniculi* antibodies among different regions are probably attributable to different rabbit breeds, sample capacities, time of investigations, and testing methods, as well as geographical and ecological factors.

The serum positive rate for *T. gondii* in the current work was lower than that of *E. cuniculi*, which was consistent with previous literature reports [13,14]. The spreading of *E. cuniculi* among herds was remarkably promoted by directly transmitting spores from urine of affected animals [14].

In the present study, the highest seroprevalence of *T. gondii* was found in New Zealand rabbits, while Rex rabbits had the highest seroprevalence of *E. cuniculi.* Regarding the breed of rabbits, de Lima *et al.* [5] and Alvarado-Esquivel *et al.* [3] reported a higher seroprevalence of *T. gondii* in New Zealand rabbits than in other breeds. Pan *et al.* also revealed higher serum positive rates for *E. cuniculi* among Rex rabbits than those among New Zealand and Japanese White Rabbits (*p* < 0.01) [15]. These results indicate that there may be a potential association between the breed of rabbits and the seropositivity against *T. gondii* and *E. cuniculi*. Most epidemiological surveys indicated no association between *T. gondii* and *E. cuniculi* infection and gender of the animals [13,18,19]. In our study, the gender of rabbits was not a significant risk factor for the presence of infection with *T. gondii*, which was in agreement with many previous studies [5,18]. However, female rabbits exhibited remarkably higher seroprevalence for *E. cuniculi* in comparison to male animals of our study. The role of gender in the epidemiology of rabbit encephalitozoonosis requires further research.

Increasing age was confirmed as a risk factor for both *T. gondii* and *E. cuniculi* infection. The observed higher seroprevalence for both protists in older rabbits suggests that these infections are mainly maintained by horizontal rather than vertical transmission. This finding is in accordance with other similar surveys [13,17].

Rabbits fed with fruits, vegetables and grains had the highest serum positive rates of *T. gondii* and *E. cuniculi* according to the current study, which was consistent with other reports [3]. This might be because these foods had a higher level of contamination of *T. gondii* oocysts or *E. cuniculi* spores than feed or mixed food. Cats play an essential role in *T. gondii* spreading since they are the final hosts and shed oocysts into the environment [11,20]. In this study, the presence of cats on rabbit farms was confirmed as one of the risk factors for occurrence of *T. gondii* infection among rabbits, which accorded with findings in previous literature reports [5,9].

In terms of rearing systems, both *T. gondii* and *E. cuniculi* seroprevalence rates were significantly higher in rabbits raised on household farms than in those raised on commercial farms, which is probably due to lower hygiene standards on household farms. Our findings are similar to those of previous reports [12,14]. These results suggest that the rearing system is a very important risk factor associated with *T. gondii* infection in rabbits as well as *E. cuniculi* infection.

## Conflict of interest statement

The authors declare that they have no conflicts of interest in relation to this article.
